# Prevalence patterns and associated factors of psychological distress among Syrian refugees in middle eastern host countries: a scoping review

**DOI:** 10.3389/fpsyg.2026.1807647

**Published:** 2026-06-08

**Authors:** Saleh Khataybeh, Mahadir Ahmad, Fawwaz Momani

**Affiliations:** 1 Clinical Psychology & Behavioural Health Programme, National University of Malaysia, Kuala Lumpur, Malaysia; 2 Department of Counseling Psychology, Yarmouk University, Irbid, Jordan

**Keywords:** anxiety, post-migration stressors, psychological distress, PTSD, scoping review, social support, Syrian refugees

## Abstract

Since 2011, the Syrian conflict has generated one of the largest displacement crises globally, with most Syrian refugees hosted in neighboring Middle Eastern countries. These host settings differ substantially in legal, social, settlement, and service conditions, which shapes both refugee experiences and the interpretation of mental health evidence. Protracted displacement, cumulative trauma, and post- migration adversity have contributed to a substantial burden of psychological distress, yet distress may be expressed not only through post-traumatic stress, depression, and anxiety symptoms, but also through culturally embedded experiences such as somatic complaints, grief, family disruption, role loss, stigma, uncertainty, and reduced dignity. This scoping review mapped prevalence patterns of psychological distress and associated risk and protective factors among Syrian refugees residing in Middle Eastern host countries, including Jordan, Lebanon, Turkey, Iraq, and Egypt. Following the Arksey and O’Malley framework and reported in accordance with PRISMA ScR, we searched PubMed, Web of Science Core Collection, and Scopus for peer-reviewed English language studies published between 2011 and 2025, with searches last updated in December 2025. Records were screened at title and abstract and full text stages using predefined criteria. Screening and data extraction were conducted by a single reviewer using standardized forms, with decisions and extracted data reviewed and discussed with a second author to enhance consistency. Eighteen studies met inclusion criteria, most conducted in Jordan, Lebanon, and Turkey, with fewer in Iraq and Egypt. Psychological distress was consistently substantial, most commonly reflected in elevated post-traumatic stress, depressive, and anxiety symptoms, while reported prevalence patterns varied widely by host country context, living setting, subgroup, sampling frame, and instruments or cut offs. Distress was repeatedly associated with trauma exposure and post-migration living difficulties, including economic hardship, legal insecurity, and daily stressors, whereas social connectedness and perceived support emerged as protective correlates. Findings highlight the need for scalable, culturally responsive psychosocial interventions and service models that reduce structural barriers to access, address stigma, and account for culturally embedded expressions of distress in protracted displacement settings.

**Systematic review registration:**

https://osf.io/vmju8/overview

## Introduction

The Syrian conflict, which erupted in 2011, has generated one of the largest displacement crises globally, forcing millions of Syrians to flee their homes, including more than 7.4 million internally displaced people and over 5.5 million registered refugees in neighboring countries (UNHCR, 2025). Middle Eastern host countries, particularly Turkey, Lebanon, and Jordan, have absorbed the majority of displaced Syrians, placing sustained pressure on health systems, labor markets, and social infrastructure (Wells et al., 2016; Hendrickx et al., 2020). However, these host countries do not represent a uniform displacement context; they differ in legal protections, registration systems, access to work, camp and non-camp settlement patterns, and availability of health and psychosocial services. Beyond material deprivation, the psychological consequences of protracted displacement have been substantial. Syrian refugees have often experienced multiple, cumulative stressors, including war-related violence, family separation, economic hardship, discrimination, and ongoing uncertainty regarding legal status and future prospects (Alpak et al., 2015; Kazour et al., 2017).

Refugee mental health is frequently discussed through the lens of psychological distress, a transdiagnostic construct capturing co-occurring symptoms of depression, anxiety, and post-traumatic stress and closely linked to impaired functioning and reduced well-being (Steel et al., 2009; Turrini et al., 2017). However, in Syrian refugee populations, psychological distress should not be understood only through standardized psychiatric symptom categories. Suffering may also be experienced and communicated through culturally embedded expressions, including somatic complaints, grief, loss of social and family roles, dependency, family disruption, stigma, uncertainty, and reduced dignity in displacement. Evidence suggests that distress reflects the combined effects of pre-migration trauma and post-migration stressors, including chronic insecurity, disrupted roles, daily living difficulties, and limited opportunities for recovery (Hendrickx et al., 2020; Porter and Haslam, 2005; Miller and Rasmussen, 2011). In Middle Eastern host-country settings, poverty, legal precarity, and stigma surrounding mental illness can further compound risk by limiting help-seeking, continuity of care, and engagement with psychosocial supports, underscoring the need for context-sensitive and scalable responses (Wells et al., 2016; Thornicroft et al., 2007; Dangmann et al., 2022).

Although mental health outcomes among refugees have been widely studied, evidence on Syrian refugees residing in Middle Eastern host countries remains fragmented across settings, population subgroups, and methodological approaches. Variability in study designs, outcome measures, host-country conditions, and contextual focus limits coherent conclusions regarding prevalence patterns of psychological distress and associated factors across the region (Hendrickx et al., 2020; Fazel et al., 2012). This fragmentation also hinders identification of consistent and modifiable risk and protective processes, such as daily stressors, social connectedness, coping resources, family support, and barriers to service access that can inform scalable psychosocial interventions. Moreover, existing review-level evidence has often emphasized high-income resettlement contexts or summarized outcomes without systematically accounting for post-migration living conditions in neighboring host states (Turrini et al., 2017; Reed et al., 2012). Mapping prevalence patterns alongside modifiable risk and protective factors within Middle Eastern host-country contexts is therefore needed, particularly given heterogeneity in measurement tools, cut-offs, cultural interpretability of distress, and living contexts, including camp versus non-camp/community settings.

Accordingly, this scoping review aims to map and synthesize empirical evidence on (a) reported prevalence patterns of psychological distress, including depression, anxiety, post-traumatic stress symptoms, and related distress presentations, and (b) associated risk and protective factors among Syrian refugees residing in Middle Eastern host countries. The review follows the Arksey and O’Malley framework and adheres to PRISMA-ScR guidelines, charting outcomes alongside measurement approaches, living-context characteristics, and post-migration conditions to identify intervention-relevant priorities and evidence gaps in protracted displacement settings (Arksey and O’Malley, 2005; Tricco et al., 2018).

## Materials and methods

### Study design and reporting

This scoping review mapped the extent, range, and nature of empirical research on the prevalence and associated factors of psychological distress among Syrian refugees residing in Middle Eastern host countries. The review followed the Arksey and O’Malley framework and is reported in accordance with the PRISMA-ScR reporting guidelines (Arksey and O’Malley, 2005; Tricco et al., 2018).

### Protocol

A review protocol was developed *a priori*. The protocol is publicly available on the Open Science Framework (OSF)^1^.

### Research questions

The review addressed the following questions:

*RQ1*: How has psychological distress among Syrian refugees been reported and measured across Middle Eastern host countries, and what ranges and patterns of estimates are reported across studies?

*RQ2*: What individual, interpersonal-, and structural-level risk and protective factors have been associated with psychological distress in this population, and what intervention-relevant evidence gaps emerge for host-country settings?

### Eligibility criteria

Studies were included if they:

- Were peer-reviewed journal articles published between 2011 and 2025 (inclusive);
- Included Syrian refugees residing in Middle Eastern host countries (Jordan, Lebanon, Turkey, Iraq, and Egypt);
- Assessed psychological distress or closely related mental health outcomes (e.g., depressive symptoms, anxiety symptoms, post-traumatic stress symptoms, and related distress presentations) using a standardized quantitative instrument and/or a structured clinical assessment. For studies contributing prevalence estimates, outcomes had to be reported as a proportion meeting a study-defined threshold (scale cut-off or clinical diagnosis). When a study reported a prevalence proportion but did not explicitly state the cut-off/threshold in the manuscript, the estimate was retained and the cut-off was recorded as “NR” (not reported); and.
- Reported sufficient methodological and outcome information to allow extraction of measurement details (instrument and cut-off/diagnostic criteria, where applicable) and either (i) prevalence estimates (%, and n/N when available) or (ii) associated risk/protective factors relevant to the review aims.

Studies were excluded if they:

- Were conducted inside Syria, in resettlement contexts outside the specified Middle Eastern host countries (Jordan, Lebanon, Turkey, Iraq, and Egypt), or otherwise outside the defined host-country setting;
- Focused on non-refugee populations, did not clearly identify participants as Syrian refugees, including host-community clinical samples not identified as Syrian refugees, or addressed crises unrelated to the Syrian conflict;
- Were non-peer-reviewed materials, such as editorials, conference abstracts, or commentaries;
- Were review articles, such as systematic reviews, meta-analyses, or scoping reviews; these were used only for citation tracking and contextual interpretation; or.
- Did not provide extractable data relevant to the review aims, that is, lacked a defined measurement approach and either a prevalence estimate based on a stated threshold/diagnostic criterion or information on associated factors.

Quantitative evidence informed the mapping of reported estimates and the synthesis of associated factors.

### Data extraction

We defined prevalence as the proportion of participants meeting a study-defined threshold for each outcome, based on a scale cut-off or clinical diagnosis. Prevalence estimates were extracted as percentages and, when available, as n/N, alongside the instrument and cut-off used. Extracted estimates were summarized by host country, setting, outcome domain, instrument, and cut-off (Table 1). Where studies reported only continuous symptom scores without a defined case threshold, they were retained for descriptive mapping of associated factors but were not included in the prevalence tabulation.

**Table 1:** Summary of included studies on psychological distress among Syrian refugees in Middle Eastern host countries (*n* = 18).

No.	Study	Host country	N	Population/setting	Instrument/cut-off	Outcome	Prevalence (%)	Study design	DOI
1	Kazour et al. (2017)	Lebanon	452	Household/community sample	MINI structured diagnostic interview; DSM-IV diagnosis-based assessment	Current PTSD	27.2	Household cross-sectional	10.1016/j.comppsych.2016.09.007
2	Basheti et al. (2019)	Jordan	186	Pharmacist-led refugee sample	HTQ-16; mean > 2.5	Probable PTSD	38.7	Pharmacist-led cross-sectional	10.18549/PharmPract.2019.3.1475
3	Acarturk et al. (2021)	Turkey	1,678	Population/community sample	HSCL-25, cut-off NR; PCL-5 ≥ 33	Depression/anxiety/PTSD	34.7/36.1/19.6	Population cross-sectional	10.1007/s00127-020-01941-6
4	Scherer et al. (2020)	Turkey	852	Children/adolescents; population-based sample	CRIES-8, cut-off NR; CES-DC, cut-off NR; SCARED, cut-off NR	Depression/PTSD/anxiety	12.5/11.5/9.2	Population-based survey	10.1017/S2045796020001079
5	Mahmood et al. (2019)	Iraq, Kurdistan Region	988	Camp-based refugee sample	PCL-5, cut-off NR; HSCL-25, cut-off NR	PTSD/depression	60.0/59.4	Camp cross-sectional	10.1186/s13031-019-0238-5
6	Acarturk et al. (2018)	Turkey	781	Camp-based refugee sample	IES-R ≥ 33; BDI, cut-off NR	PTSD/depression	83.4/37.4	Camp cross-sectional	10.1097/NMD.0000000000000693
7	Alpak et al. (2015)	Turkey	352	Camp-based refugee sample	HTQ, cut-off NR; HSCL-25, cut-off NR	PTSD/depression	33.5/37.4	Camp cross-sectional	10.1016/j.comppsych.2014.11.011
8	Abougazia et al. (2025)	Egypt	420	NGO community-center sample	BDI-II, cut-off NR; PCL-C, cut-off NR; SWLS, no clinical cut-off	Depression/PTSD	15.2/89.3	Cross-sectional	10.21608/jhiph.2025.398471.1198
9	Poole et al. (2018)	Lebanon	827	Household/community sample	MINI structured diagnostic interview; DSM-IV MDE and PTSD modules; diagnosis-based assessment	Major depression/PTSD	43.9/27.2	Household cross-sectional	10.1016/j.jad.2018.03.015
10	Sabry et al. (2020)	Egypt	94	Working refugees; convenience sample	BDI, cut-off NR; Taylor Anxiety Scale, cut-off NR	Depression/anxiety	63.0/89.0	Cross-sectional	10.21608/ejcm.2020.68625
11	McGrath et al. (2020)	Turkey	1,444	Community sample	PHQ-15 ≥ 10	Somatic distress	41.3	Community cross-sectional	10.1016/j.jpsychores.2020.109993
12	Brooks et al. (2021)	Jordan	507	Women attending clinics	CES-D ≥ 4; GAD-7 ≥ 10; PCL-5 ≥ 23	Depression/anxiety/PTSD	62.9/57.5/66.2	Clinic cross-sectional	10.1186/s12905-021-01584-y
13	Clark et al. (2021)	Lebanon	230	Sexual minority and transgender refugee sample; respondent-driven sampling	CES-D ≥ 16; BAI ≥ 26; PCL-C ≥ 50	Depression/anxiety/PTSD	63.0/21.3/33.0	Cross-sectional, RDS	10.1136/bmjopen-2020-046996
14	McEwen et al. (2023)	Lebanon	1,591	Refugee children in informal settlements	MINI-KID structured diagnostic interview; DSM-5 diagnosis-based assessment	PTSD/anxiety/depression/ODD	39.6/47.8/20.1/26.9	Prospective cohort	10.1038/s44220-023-00017-z
15	Ibrahim and Hassan (2017)	Iraq, Kurdistan Region	91	Camp-based refugee sample	HTQ; mean score > 2.5	PTSD symptoms	35–38	Camp cross-sectional	10.3389/fpsyg.2017.00241
16	Al-Smadi et al. (2020)	Jordan	1,200	Population-based refugee sample	K10 ≥ 20	Severe psychological distress	44.7	Population cross-sectional	10.1371/journal.pone.0232214
17	Ruhnke et al. (2024)	Turkey	620	Cross-sectional refugee sample	PHQ-8 continuous score; no prevalence cut-off reported	Depression symptoms	39.5	Cross-sectional	10.1016/j.socscimed.2024.116700
18	Stevenson et al. (2019)	Lebanon	35	Maternal/clinic sample	EPDS ≥ 13	Maternal depression	75.0	Pilot clinic cross-sectional	10.1038/s41598-019-48247-5

BAI, beck anxiety inventory; BDI, beck depression inventory; CES-D, center for epidemiologic studies depression scale; CES-DC, center for epidemiological studies depression scale for children; CRIES-8, children’s revised impact of event scale; EPDS, Edinburgh postnatal depression scale; GAD-7, Generalized anxiety disorder-7; HSCL-25, Hopkins symptom checklist-25; HTQ, Harvard trauma questionnaire; IES-R, Impact of event scale-revised; K10, Kessler psychological distress scale; MDE, major depressive episode; MINI, Mini international neuropsychiatric interview; MINI-KID, Mini international neuropsychiatric interview for children and adolescents; NR, not reported; ODD, oppositional defiant disorder; PCL-5, PTSD checklist for DSM-5; PCL-C, PTSD Checklist-civilian version; PHQ-8/PHQ-15, patient health questionnaire-8/15; PTSD, post-traumatic stress disorder; RDS, respondent-driven sampling; SCARED, screen for child anxiety related emotional disorders; SWLS, satisfaction with life scale. Reported estimates reflect study defined instruments, thresholds, diagnostic criteria, sampling frames, and population subgroups. Therefore, prevalence estimates should not be interpreted as directly comparable across host countries, living settings, or subgroups. Some studies did not report cut off thresholds, and some used continuous symptom scores or structured diagnostic interviews rather than scale based cut offs. These differences were considered when interpreting prevalence patterns descriptively rather than as pooled or directly comparable estimates.

To address contextual and subgroup variation, we also extracted information on host-country setting, living context, sampling frame, and population subgroup, including camp-based, non-camp/community, clinic-based, population-based, women-only, child/adolescent, maternal, working-refugee, and other specialized samples where reported. When included studies reported formal statistical comparisons, association tests, effect estimates, confidence intervals, or *p*-values for risk/protective factors or subgroup differences, these were extracted and charted. However, no new inferential statistical tests were conducted across studies because the included studies differed substantially in design, sampling frames, instruments, cut-off thresholds, reported outcomes, and availability of n/N data.

### Information sources and search strategy

The search strategy was guided by the Population–Concept–Context (PCC) framework, and free-text keywords were combined with database-specific controlled vocabulary where applicable, such as MeSH terms in PubMed. Search concepts captured: (1) Syrian refugees or asylum seekers; (2) psychological distress and related mental health outcomes, including depression, anxiety, and post-traumatic stress; and (3) Middle Eastern host settings, including Middle East, Jordan, Lebanon, Turkey, Iraq, and Egypt. Boolean operators (AND/OR), phrase searching, and truncation were tailored to each database to optimize sensitivity and specificity.

Peer-reviewed English-language studies were identified through searches in PubMed (MEDLINE), Web of Science Core Collection, and Scopus, with searches last updated in December 2025. The full electronic search strategies for each database, including all keywords, controlled vocabulary, and applied limits, are provided in Supplementary Table S1. All retrieved records were exported to Zotero for reference management and de-duplication before screening. In addition, reference lists of included studies and relevant review articles were hand-searched to identify further eligible publications.

### Study selection

Following de-duplication in Zotero, records were screened in two stages: (1) title and abstract screening and (2) full-text assessment for eligibility, using the predefined eligibility criteria. Screening and data extraction were conducted by a single reviewer using predefined eligibility criteria and a standardized extraction form. A second author (MA) reviewed eligibility decisions in cases of uncertainty and discussed the extracted data to ensure consistency, accuracy, and interpretability. Uncertain records were re-checked before final inclusion decisions were made. At the full-text stage, seventeen articles were excluded; reasons for exclusion were recorded and summarized in the PRISMA-ScR flow diagram. The overall study selection process, including records identified via reference-list screening/citation tracking, was documented using a PRISMA-ScR flow diagram.

### Data charting and extraction

Data were charted using a standardized extraction form in Excel. For each included study, we extracted: (a) bibliographic details, including author and year; (b) host country and living context, such as camp versus non-camp/community, where reported; (c) sample characteristics, including population subgroup, age group, sex/gender where available, sample size, and study design/sampling approach; (d) psychological distress outcomes assessed, such as depression, anxiety, post-traumatic stress symptoms, and related distress outcomes; (e) measurement approach, including instrument(s) used and any reported cut-off thresholds or diagnostic criteria; (f) reported prevalence estimates/patterns, when applicable; (g) associated risk and protective factors, grouped across individual, interpersonal, and structural/contextual domains; and (h) reported statistical evidence for associations or subgroup comparisons, including statistical tests, effect estimates, confidence intervals, and *p*-values where available.

Data charting and extraction were conducted by a single reviewer using a predefined, standardized charting form, with verification against full texts to ensure accuracy and consistency. Extracted data were reviewed and discussed with a second author (MA) to enhance consistency and accuracy.

### Critical appraisal

Consistent with the primary purpose of a scoping review, to map the breadth and characteristics of the evidence rather than to generate pooled effect estimates, and in line with methodological guidance for scoping reviews, we did not conduct a formal risk-of-bias assessment of included studies. This approach is consistent with PRISMA-ScR guidance, which emphasizes evidence mapping rather than formal quality appraisal or effect estimation (Arksey and O’Malley, 2005; Tricco et al., 2018). Instead, to support interpretation and applied relevance, we charted key methodological and measurement features likely to contribute to heterogeneity across studies, including study design, sampling approach, setting, population subgroup, and the measurement approach for psychological distress, including instrument(s) used and any reported cut-off thresholds/diagnostic criteria. These indicators were used to contextualize variability in reported prevalence patterns and associations across host-country settings.

### Data synthesis

Extracted data were synthesized descriptively to align with the scoping review objectives. Findings were summarized using a structured narrative synthesis, focusing on prevalence patterns, reported ranges and distribution of estimates, and variation by host country, living context, population subgroup, sampling frame, and measurement approach where reported. Associated risk and protective factors were charted and summarized across individual, interpersonal, and structural/contextual domains and interpreted in light of measurement and contextual heterogeneity.

Where available, statistical results reported in the included studies, including *p*-values, effect estimates, and confidence intervals, were summarized descriptively to support interpretation of associations between psychological distress and relevant factors. Formal statistical testing comparing prevalence across host countries, living settings, or subgroups was not conducted at review level because the studies were not sufficiently comparable in design, sampling, outcome definitions, measurement instruments, cut-off thresholds, and reporting completeness. Therefore, host-country, setting, and subgroup patterns were interpreted cautiously as descriptive evidence patterns rather than direct statistical comparisons. Results were presented to highlight consistent patterns, sources of variability linked to instruments and cut-offs, and intervention-relevant evidence gaps across Middle Eastern host-country settings.

## Results

### Study selection results

**Figure 1:**
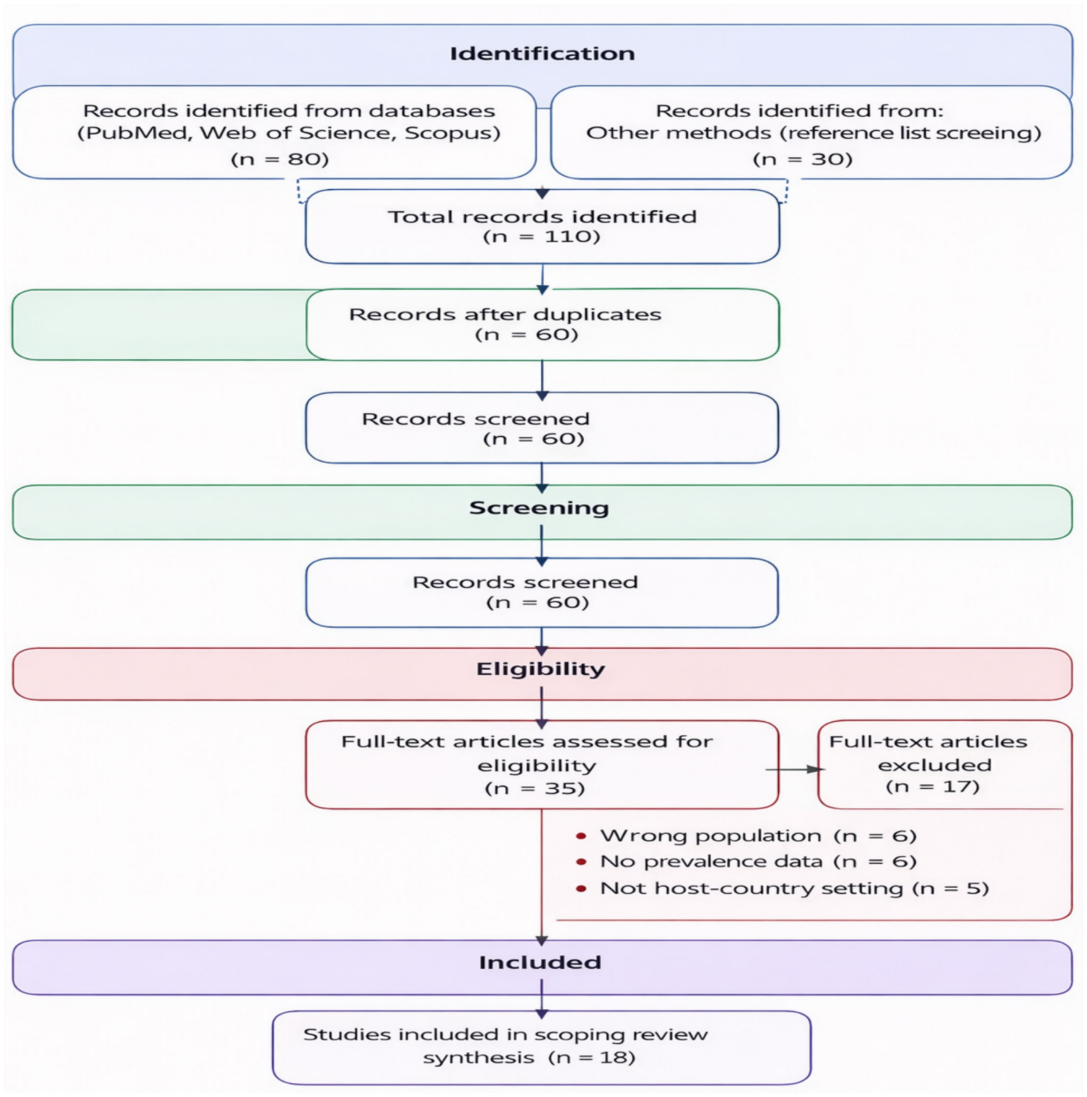
PRISMA-ScR flow diagram of the study selection process (*n* = 18).

The database searches identified 80 records, with an additional 30 records identified through reference-list screening (citation tracking). After de-duplication, 60 records remained for title and abstract screening, of which 25 were excluded. Thirty-five full-text articles were assessed for eligibility, and 17 were excluded at the full-text stage, with reasons recorded and reported in the PRISMA-ScR flow diagram. In total, 18 studies were included in the scoping review synthesis (Figure 1).

### Characteristics of included studies

The 18 included studies were heterogeneous in design, participant characteristics, living contexts, sampling frames, and measurement approaches. Most studies used cross-sectional designs, with fewer longitudinal or prospective designs. Samples spanned key subgroups, including adults, women, children/adolescents, maternal samples, working refugees, family units, and other specialized or service-linked groups. Participants were drawn from camp-based, non-camp/community, clinic-based, service-based, and population-based settings. Psychological distress was operationalized using a range of instruments and cut-off thresholds/diagnostic criteria, which likely contributed to between-study variability in reported prevalence patterns and limited direct comparability across settings. An overview of study characteristics and measurement approaches is provided in Table 1.

### Geographical distribution

The included evidence was concentrated in the major Middle Eastern host countries—particularly Jordan, Lebanon, and Turkey—with comparatively limited study coverage in Iraq and Egypt. This geographical clustering likely reflects both the regional distribution of Syrian refugee populations and differences in research infrastructure, service delivery models, legal and policy environments, and access constraints across host settings. Because Syrian refugees in these countries live under different legal, social, service, and settlement conditions, host-country differences in prevalence should be interpreted cautiously and should not be treated as direct comparisons between equivalent refugee populations. The imbalance also indicates evidence gaps in under-studied host-country contexts, which may limit the transferability of reported prevalence patterns and associated factors and have implications for prioritizing context-sensitive psychosocial intervention planning across the region.

### Mental health outcomes and measurement approaches

Across the included studies, psychological distress was operationalized primarily through symptoms of post-traumatic stress and depression, with anxiety commonly assessed as a co-occurring outcome. Some studies also assessed related outcomes, such as somatic distress, maternal depression, severe psychological distress, or broader psychiatric morbidity. Studies relied on a range of standardized instruments and, in some cases, diagnostic approaches, with variation in scoring methods, language adaptation, and cut-off thresholds across settings and subgroups. This heterogeneity in measurement approach, alongside differences in sampling frames and living contexts, likely contributed to variability in reported prevalence patterns and limited direct comparability across studies. Accordingly, findings are interpreted as evidence patterns rather than as a single consolidated prevalence estimate, and measurement differences are highlighted as an applied priority for both surveillance and intervention evaluation in host-country settings.

### Prevalence patterns

Overall, the included studies indicated substantial psychological distress among Syrian refugees across Middle Eastern host countries, with prevalence estimates varying by outcome, host country, population subgroup, living context, sampling frame, and measurement approach. Across outcomes, PTSD, depression, and anxiety-related symptom prevalence showed wide dispersion, consistent with heterogeneity in instruments and cut-off thresholds/diagnostic criteria and differences in sampling frames. Higher prevalence estimates were more frequently reported in camp-based samples, clinic or service-based samples, women-only or maternal samples, and among participants with greater trauma exposure and higher levels of post-migration adversity. However, these patterns should be interpreted cautiously because the included studies differed substantially in design, sampling methods, population composition, living context, and outcome definitions. Given this variability, prevalence is summarized as ranges and patterns rather than as a pooled estimate or formal host-country comparison. Study-level outcome measures and reported prevalence information, where available, are summarized in Table 1 to support transparent cross-study comparison while acknowledging measurement and contextual heterogeneity.

### Factors associated with psychological distress

Across the included studies, factors associated with psychological distress clustered into interrelated, multi-level domains with clear applied relevance. At the pre-migration level, conflict-related trauma exposure, including violence, loss, and forced displacement, was consistently associated with higher distress, most notably post-traumatic stress and depressive symptoms. At the post-migration level, ongoing living difficulties, including economic hardship, unemployment, legal insecurity, uncertainty about the future, dependency, and daily stressors, were frequently linked to poorer mental health outcomes. These findings indicate that distress is sustained not only by past trauma but also by current adversity and prolonged insecurity in host-country contexts.

Social and structural conditions further shaped distress patterns. Lower perceived social support, social isolation, and reduced community connectedness were commonly associated with higher distress, whereas stronger family and community support functioned as protective correlates. In parallel, mental health stigma and structural barriers to care, including cost, limited availability, and access constraints, were repeatedly noted as factors that may amplify distress and reduce help-seeking and service engagement. Several subgroups were consistently identified as more vulnerable, particularly women and children/adolescents, highlighting the importance of stratifying interpretation by subgroup and prioritizing targeted, context-sensitive psychosocial responses for groups facing compounded developmental, gendered, social, and structural stressors. Where included studies reported statistical tests, effect estimates, confidence intervals, or *p*-values for associated factors or subgroup differences, these were charted and summarized descriptively rather than re-analyzed as pooled or direct comparative estimates.

Table 2 summarizes the risk and protective factors reported across the included studies. Where original studies reported statistical associations, these were charted descriptively rather than pooled or re-tested. Because the included studies differed substantially in design, sampling frame, setting, subgroup composition, measurement instruments, and cut-off thresholds, the reported associations should be interpreted as evidence patterns rather than direct comparative estimates.

**Table 2:** Reported statistical evidence for risk and protective factors associated with psychological distress among Syrian refugees in included studies.

No.	Study	Host country	Population/setting	Outcome	Reported associated factors	Reported statistical evidence	Interpretation
1	Kazour et al. (2017)	Lebanon	Household/community sample	Current PTSD	War exposure, traumatic events, displacement related stressors, and social adversity	PTSD was assessed using a structured diagnostic interview. Trauma related exposures and displacement stressors were reported as associated with PTSD morbidity; exact test statistics to be verified from source article.	Higher trauma and displacement related adversity were associated with greater PTSD burden.
2	Basheti et al. (2019)	Jordan	Pharmacist led refugee sample	Probable PTSD	Age, education, smoking status, trauma exposure, gender, and PTSD symptom severity	Age group: *χ^2^* = 8.96, *p* < 0.01; education: *χ^2^* = 12.92, *p* = 0.012; smoking status: *χ^2^* = 12.0, *p* = 0.002; experiencing trauma: *χ^2^* = 11.03, *p* = 0.001; gender and HTQ 16 severity: *t*(184) = 2.161, *p* = 0.032; age correlation with HTQ 16: *r* = 0.273, *p* < 0.001; gender correlation: *r* = 0.145, *p* = 0.049; smoking correlation: *r* = 0.257, *p* < 0.001; logistic regression model: *χ^2^*(df = 4) = 25.05, *p* < 0.001.	Older age, lower education, smoking, trauma exposure, and gender were associated with probable PTSD or higher PTSD symptom severity.
3	Acarturk et al. (2021)	Turkey	Population/community sample	Depression, anxiety, PTSD	Female sex, age, trauma exposure, post migration stressors, and lower social support	Multivariable regression identified trauma exposure and post migration stressors as risk factors for common mental disorders, while social support showed a protective association; exact ORs, CIs, and *p* values to be verified from source article.	Psychological symptoms were associated with both prior trauma and current social or living difficulties.
4	Scherer et al. (2020)	Turkey	Children/adolescents; population based sample	Depression, PTSD, anxiety	Age, gender, household poverty, education, household size, and family adversity	Group comparisons and regression analyses showed variation in mental health outcomes by age, gender, poverty, education, and household characteristics; exact test statistics to be verified from source article.	Child and adolescent distress was patterned by developmental, family, and socioeconomic factors.
5	Mahmood et al. (2019)	Iraq, Kurdistan Region	Camp based refugee sample	PTSD and depression	Trauma exposure, cumulative traumatic events, camp related hardship, and displacement related adversity	Regression and association analyses identified trauma exposure and displacement related adversity as correlates of PTSD and depression symptoms; exact coefficients and p values to be verified from source article.	Greater cumulative adversity was associated with higher PTSD and depression.
6	Acarturk et al. (2018)	Turkey	Camp based refugee sample	PTSD and depression	Female sex, previous mental health problems, life threat, injury of a loved one, torture of a loved one, and dissatisfaction with camp conditions	Predictors included female sex for PTSD, OR = 4.1; previous mental health problems for PTSD, OR = 4.5; life threat for PTSD, OR = 3.0; injury of a loved one for PTSD, OR = 1.8; female sex for depression, OR = 5.1; previous mental health problems for depression, OR = 2.9; loved one tortured for depression, OR = 1.7; dissatisfaction with camp for depression, OR = 1.7.	Gender, prior mental health problems, trauma exposure, and dissatisfaction with camp conditions were associated with higher PTSD and depression.
7	Alpak et al. (2015)	Turkey	Camp based refugee sample	PTSD and depression	War related trauma, family loss or separation, and displacement related hardship	Associations were reported between traumatic war experiences, family disruption, and PTSD/depressive symptoms; exact statistical values to be verified from source article.	War related trauma and family disruption were associated with poorer mental health.
8	Abougazia et al. (2025)	Egypt	NGO community center sample	Depression, PTSD, life satisfaction	Trauma exposure, economic stressors, social stressors, and satisfaction with life	Association testing linked psychological symptoms with trauma, socioeconomic stressors, and life satisfaction indicators; exact *p* values to be verified from source article.	Higher stress exposure was associated with higher depression and PTSD, while better life satisfaction was protective.
9	Poole et al. (2018)	Lebanon	Household/community sample	Major depression and PTSD	Poverty, household adversity, trauma exposure, and post migration stressors	Regression analyses identified socioeconomic hardship and trauma related variables as risk factors for major depression and PTSD; exact ORs, CIs, and p values to be verified from source article.	Economic and household adversity contributed to psychiatric morbidity.
10	Sabry et al. (2020)	Egypt	Working refugees; convenience sample	Depression and anxiety	Work related hardship, economic insecurity, displacement stressors, and social conditions	Statistical comparisons examined depression and anxiety symptoms across sociodemographic and living difficulty variables; exact test statistics to be verified from source article.	Working refugees experienced high psychological symptom burden linked to socioeconomic stressors.
11	McGrath et al. (2020)	Turkey	Community sample	Somatic distress	Female sex, chronic disease or disability, family mental health problems, depression, anxiety, PTSD, household economic situation, and education	High somatic distress was higher among females: *χ^2^*(1) = 66.25, *p* < 0.001; depression: *χ^2^*(1) = 304.76, *p* < 0.001; anxiety: *χ^2^*(1) = 405.43, *p* < 0.001; PTSD: *χ^2^*(1) = 155.46, *p* < 0.001. Multivariable ordered logistic regression showed female sex OR = 2.91, 95% CI 2.32 to 3.63, *p* < 0.001; chronic disease or disability OR = 2.87, 95% CI 2.28 to 3.61, *p* < 0.001; family member with mental health problems OR = 1.65, 95% CI 1.30 to 2.09, *p* < 0.001; depression OR = 3.05, 95% CI 2.32 to 4.01, *p* < 0.001; anxiety OR = 4.64, 95% CI 3.50 to 6.15, *p* < 0.001; PTSD OR = 1.75, 95% CI 1.28 to 2.39, *p* < 0.001; average household economic situation OR = 0.64, 95% CI 0.52 to 0.80, *p* < 0.001; good or very good economic situation OR = 0.53, 95% CI 0.31 to 0.88, *p* = 0.015; years of education OR = 0.95, 95% CI 0.93 to 0.98, *p* = 0.001.	Somatic distress was strongly associated with gender, physical health, family mental health history, psychiatric symptoms, poorer economic conditions, and lower education.
12	Brooks et al. (2021)	Jordan	Women attending clinics	Depression, anxiety, PTSD	Trauma exposure, gendered vulnerability, clinic linked stressors, social adversity, and support factors	Statistical analyses examined associations between mental health outcomes and trauma, demographic, and social variables; exact statistical values to be verified from source article.	Women attending clinics showed high symptom burden linked to trauma and social adversity.
13	Clark et al. (2021)	Lebanon	Sexual minority and transgender refugee sample; RDS	Depression, anxiety, PTSD	Displacement status, income, relationship status, number of locations lived in Lebanon, legal status, sexual minority discrimination, sexual minority assault, internalised sexual minority stigma, and internalised Syrian stigma	Compared with Lebanese participants, displaced Syrian participants had higher depression, *p* < 0.001; higher anxiety, *p* < 0.05; and higher PTSD, *p* < 0.001. Among displaced Syrian MSM and transgender women, adjusted models showed associations for income ≥ US$501 with lower depression, *p* < 0.01; lower anxiety, *p* < 0.01; and lower PTSD, *p* < 0.001. Legal status was associated with lower depression, *p* < 0.05; lower anxiety, *p* < 0.05; and lower PTSD, *p* < 0.01. Sexual minority discrimination was associated with higher depression, *p* < 0.05, and higher anxiety, *p* < 0.01. Sexual minority assault was associated with higher depression, *p* < 0.05, and higher PTSD, *p* < 0.05. Internalised Syrian stigma was associated with higher anxiety, *p* < 0.05.	Displacement related, legal, socioeconomic, and stigma related stressors were associated with psychiatric morbidity.
14	McEwen et al. (2023)	Lebanon	Refugee children in informal settlements	PTSD, anxiety, depression, ODD	Family adversity, informal settlement conditions, trauma exposure, developmental vulnerability, and household conditions	Prospective analyses examined predictors of child mental health problems across family, developmental, and settlement related domains; exact ORs, CIs, and p values to be verified from source article.	Child mental health outcomes were associated with family adversity, trauma, and informal settlement conditions.
15	Ibrahim and Hassan (2017)	Iraq, Kurdistan Region	Camp based refugee sample	PTSD symptoms	Trauma exposure, camp related hardship, displacement stressors, and demographic factors	Statistical testing examined PTSD symptoms in relation to trauma and displacement related variables; exact test statistics to be verified from source article.	Trauma and camp related hardship were associated with higher PTSD symptoms.
16	Al-Smadi et al. (2020)	Jordan	Population based refugee sample	Severe psychological distress	Social ecological determinants, individual stressors, interpersonal factors, contextual stressors, and social support	Multivariable analyses identified individual, interpersonal, and contextual determinants of mental distress; exact coefficients, CIs, and p values to be verified from source article.	Severe distress was associated with multilevel ecological stressors.
17	Ruhnke et al. (2024)	Lebanon and Turkey	Cross sectional refugee sample	Depression symptoms	Country context, access to health care, family in the same city, low education, poverty, unemployment, day labor, discrimination, and social isolation	Regression analyses identified social exclusion and discrimination as predictors in both countries. In Lebanon, health care access and family related variables were important predictors; in Turkey, low education, poverty, unemployment, and day labor were associated with depressive symptoms; exact statistical values to be verified from source article.	Social ecological determinants differed across host country contexts.
18	Stevenson et al. (2019)	Lebanon	Maternal/clinic sample	Maternal depression	Maternal vulnerability, displacement stressors, limited support, and service related barriers	Statistical analyses examined maternal depression in relation to displacement, social, and support related variables; exact test statistics to be verified from source article.	Maternal depression was high and linked to displacement related vulnerability and limited support.

PTSD, post traumatic stress disorder; HTQ 16, Harvard Trauma Questionnaire 16 item symptom scale; OR, odds ratio; CI, confidence interval; MSM, men who have sex with men; ODD, oppositional defiant disorder; RDS, respondent driven sampling. Statistical values are reported where exact values were available. For studies where original articles reported associations without uniform extractable test statistics across all factors, the statistical evidence is summarized descriptively. No new inferential testing or meta analysis was conducted because included studies differed substantially in sampling frames, settings, subgroups, instruments, outcome definitions, and cut off thresholds.

## Discussion

This scoping review mapped empirical literature on psychological distress among Syrian refugees residing in Middle Eastern host countries, synthesizing evidence on prevalence patterns and associated risk and protective factors across settings, subgroups, and measurement approaches. In this review, psychological distress is conceptualized as an umbrella construct encompassing commonly assessed mental health symptoms and syndromes among refugee populations, including depressive symptoms, anxiety symptoms, post-traumatic stress symptoms, somatic distress, and related distress outcomes. Overall, included studies indicated a substantial burden of distress, most commonly reflected in elevated post-traumatic stress, depressive, and anxiety symptoms. However, prevalence patterns varied widely, and this variability was closely linked to heterogeneity in host country contexts, living settings such as camp and community settings, population subgroups, notably women and children or adolescents, and measurement instruments and cut off thresholds or diagnostic criteria. The evidence is therefore best interpreted as identifying patterns and sources of heterogeneity rather than supporting a single consolidated prevalence estimate.

An important implication of these findings is that psychological distress among Syrian refugees should not be interpreted only through Western diagnostic categories or standardized symptom scales. Although measures of depression, anxiety, and post-traumatic stress are useful for surveillance and service planning, distress in Syrian refugee communities may also be experienced and communicated through culturally embedded forms of suffering, including somatic complaints, grief, family disruption, loss of social role, dependency, humiliation, uncertainty, and concern about social shame. This is consistent with broader refugee mental health literature showing that distress reflects both exposure to violence and the social meanings of displacement, loss, and disrupted identity (Steel et al., 2009; Porter and Haslam, 2005; Miller and Rasmussen, 2011). Therefore, prevalence estimates based on symptom scales should be understood as partial indicators of suffering rather than complete representations of refugee mental health experience.

Across the mapped literature, psychological distress was repeatedly associated with both pre migration trauma exposure and post-migration living difficulties. Trauma related to conflict, loss, and forced displacement was a consistent correlate of higher symptom burden, while ongoing post-migration adversity, particularly economic hardship, unemployment, legal insecurity, dependency, restricted opportunity, and chronic daily stressors, appeared to sustain distress over time. These findings underscore that refugee mental health in neighboring host country contexts is shaped not only by prior traumatic exposure but also by persistent structural and social conditions that constrain recovery in protracted displacement settings (Hendrickx et al., 2020; Porter and Haslam, 2005; Miller and Rasmussen, 2011). In this sense, distress should not be viewed only as an individual psychopathology problem. It is also shaped by prolonged uncertainty, restricted opportunities, unstable legal and economic conditions, and repeated threats to dignity and autonomy.

Social and interpersonal processes also emerged as central correlates of distress. Lower perceived social support, social isolation, and reduced community connectedness were commonly associated with higher distress, whereas stronger family and community support appeared protective across multiple studies. In parallel, stigma surrounding mental health and structural barriers to care, including cost, limited availability, and access constraints, were frequently reported as factors that reduce help seeking and continuity of care (Wells et al., 2016; Thornicroft et al., 2007; Dangmann et al., 2022). Together, these patterns highlight the importance of interventions that address symptoms while strengthening supportive social environments and improving pathways to accessible and culturally responsive care.

The interpretation of stigma and support also requires attention to gender, family systems, and social expectations within Syrian refugee communities. Women may experience distress through gendered burdens related to caregiving, widowhood, and dependency, exposure to violence, poverty, and fear of community judgment. Men may also experience distress when displacement disrupts provider roles, social status, and family authority. In both cases, family privacy, honor concerns, social shame around mental illness, and fear of being judged may shape how symptoms are reported and whether services are used. These dynamics may also lead some groups to underreport distress or seek help through informal family, religious, or community networks rather than formal mental health services. Therefore, intervention planning should consider the centrality of family systems, gendered role changes, and stigma as factors that shape both symptom expression and service engagement.

From an applied perspective, the mapped evidence points to intervention priorities that are both scalable and responsive to context. First, psychosocial programs should target modifiable mechanisms linked to distress in these settings, such as coping resources, emotion regulation, social connectedness, and family support, while also responding to daily stressors that are often intertwined with symptom burden. Second, service models should incorporate strategies to reduce structural barriers, including service navigation, cost reduction, outreach, and integration into primary care or community platforms, while also addressing stigma to improve engagement. Finally, subgroup differences suggest the value of targeted approaches for women and children or adolescents, including family linked and school linked models where appropriate and protection informed components for groups facing heightened vulnerability.

From a public health perspective, these findings support host country strategies that integrate scalable mental health and psychosocial support into primary care and community platforms, while addressing structural barriers such as cost, legal insecurity, and service availability that shape distress in protracted displacement settings. This approach is consistent with refugee mental health evidence showing that post-migration living conditions may be as important as prior trauma in shaping mental health outcomes (Porter and Haslam, 2005; Miller and Rasmussen, 2011; Reed et al., 2012). Therefore, psychosocial responses should not focus only on individual treatment, but should also strengthen social protection, access to services, community support, and practical assistance that reduces daily insecurity.

These findings align with broader refugee mental health literature, indicating that common mental health problems reflect the interaction of traumatic exposure and post-migration conditions, while also highlighting the distinctive realities of neighboring Middle Eastern host settings where protracted displacement and resource constraints are central (Hendrickx et al., 2020; Steel et al., 2009; Turrini et al., 2017). Evidence was concentrated in Jordan, Lebanon, and Turkey, suggesting that conclusions are disproportionately shaped by a small number of host contexts, while comparatively limited coverage in Iraq and Egypt points to important regional gaps. Host country comparisons should therefore be made cautiously because Syrian refugees in Lebanon, Jordan, Turkey, Iraq, and Egypt live under different legal, social, economic, settlement, and service conditions. Differences in camp structure, registration status, access to work, humanitarian assistance, and availability of mental health services may influence both actual distress and the likelihood that distress is detected in research.

Measurement issues are also central to interpretation. Variability in instruments and cut off thresholds or diagnostic criteria complicates cross study comparison and may partly explain the wide dispersion of reported prevalence patterns. Several studies used instruments or cut offs that were not fully reported, while others relied on continuous symptom scores or structured diagnostic interviews. In refugee research, particularly with Arabic speaking Syrian populations, it is important to consider whether instruments were translated appropriately, locally validated, and conceptually meaningful in displaced communities. Cut offs transported from other populations may not have the same meaning in Syrian refugee settings, especially when distress is expressed through somatic symptoms, grief, family role disruption, or culturally shaped idioms. Strengthening harmonization in measurement and reporting, including clearer documentation of thresholds, Arabic language validation, and context specific interpretability, would improve the utility of future surveillance and evaluation efforts.

This scoping review also highlights intervention relevant evidence gaps. First, relatively few studies employed longitudinal designs, limiting understanding of symptom trajectories and the temporal ordering of post-migration stressors and distress. Second, available quantitative evidence remains uneven across host countries and key subgroups, limiting comparability and applied interpretation across the region. Third, implementation focused research is limited, including evidence on the feasibility, acceptability, and effectiveness of scalable interventions delivered through routine community, school, or primary care platforms. Fourth, several potentially vulnerable or harder to reach groups remain underrepresented, including older adults, persons with disabilities, and people with severe mental illness, unregistered refugees, refugees who avoid formal services, and highly stigmatized groups. Addressing these gaps would support more actionable guidance for designing and targeting psychosocial responses in host country settings.

### Strengths and limitations

This review has several strengths. It applied an established scoping review framework and PRISMA ScR guidance, used systematic searching across multiple databases, and incorporated citation tracking to enhance coverage. Data were charted using a standardized extraction form and synthesized to map prevalence patterns alongside associated factors across host country contexts, subgroups, and measurement approaches, supporting an intervention relevant interpretation of the evidence landscape. The review also considered contextual features such as living setting, population subgroup, measurement instrument, and cut off threshold, which helped interpret the variability in reported prevalence patterns across studies.

Several limitations should be noted. First, the review was restricted to peer-reviewed English language studies, which may have excluded relevant evidence published in Arabic, Turkish, or other regional languages, as well as studies published in local journals or gray literature. This limitation is particularly important for Syrian refugee mental health research because regionally produced evidence may better capture local service conditions, culturally embedded expressions of distress, and context specific barriers to help seeking. Second, screening and data extraction were conducted by a single reviewer, although eligibility decisions in cases of uncertainty and extracted data were reviewed and discussed with a second author to enhance consistency. This remains a methodological limitation because independent duplicate screening and extraction were not performed for all records.

Third, consistent with scoping review objectives, a formal risk of bias assessment was not conducted; therefore, mapped associations should be interpreted cautiously and not as causal effects. Fourth, heterogeneity in sampling frames, living contexts, population subgroups, instruments, language adaptation procedures, and cut off thresholds or diagnostic criteria limited cross study comparability and precluded quantitative pooling of prevalence estimates. Several studies also did not clearly report cut off thresholds, which further limits interpretation of prevalence estimates and makes direct comparison across studies inappropriate. Finally, the geographical concentration of evidence in a small number of host settings may limit transferability to understudied contexts within the region, particularly Iraq and Egypt, and to harder to reach refugee groups who may be absent from formal service systems or research sampling frames.

## Conclusion

Syrian refugees residing in Middle Eastern host countries experience a substantial burden of psychological distress shaped by the combined effects of conflict related trauma and ongoing post-migration adversity. However, this distress should not be understood only as depression, anxiety, or post-traumatic stress symptoms. It may also be experienced and expressed through culturally embedded forms of suffering, including somatic complaints, grief, family disruption, role loss, uncertainty, stigma, dependency, and reduced dignity in displacement.

The mapped evidence indicates wide variability in reported prevalence patterns, driven by differences in host country context, living setting, population subgroup, sampling frame, and heterogeneity in measurement instruments and cut off thresholds or diagnostic criteria. Therefore, prevalence estimates should be interpreted cautiously as descriptive patterns rather than direct comparisons across countries or refugee subgroups. Applied priorities include scalable, culturally responsive psychosocial interventions that target modifiable psychological, social, and family level processes, such as coping resources, social support, family connectedness, and stigma reduction. These should be accompanied by service models that reduce structural barriers to access, including cost, legal insecurity, limited service availability, and difficulties navigating care systems. Future research should broaden coverage in understudied host settings, strengthen longitudinal and implementation focused designs, include harder to reach refugee groups, and improve harmonization in measurement, translation, validation, and reporting to support more actionable monitoring and intervention evaluation in protracted displacement contexts.

## Data Availability

The original contributions presented in the study are included in the article/supplementary material, further inquiries can be directed to the corresponding author.

## Footnotes

1
https://osf.io/vmju8/
